# TFAP2B and FOXC1 are associated with biologically and clinically distinct differentiation states in triple-negative breast cancer

**DOI:** 10.1038/s41598-026-68832-9

**Published:** 2026-09-11

**Authors:** Franz-Leonard Klaus, Isabella Kitzke, Evelyn Klein, Marion Kiechle, Carolin Mogler, Alexander Muckenhuber

**Affiliations:** 1 Institute of Pathology, TUM School of Medicine and Health, TUM, Munich, Germany; 2 Department of Gynecology and Obstetrics, TUM School of Medicine and Health, TUM University Hospital, Klinikum Rechts der Isar, Munich, Germany

**Keywords:** Cancer, Oncology

## Abstract

Triple-negative breast cancer (TNBC) comprises biologically distinct subtypes, including luminal androgen receptor (LAR) tumors, but routine immunohistochemical markers incompletely capture lineage-associated differentiation states. We investigated TFAP2B, an AP-2 family transcription factor linked to epithelial differentiation, and FOXC1, a basal-like-associated transcription factor, as complementary markers of luminal and basal differentiation in TNBC. In a tissue microarray of 105 TNBCs, tumors were stratified as TFAP2B-dominant, FOXC1-dominant, or double-negative according to relative marker predominance and characterized using lineage, proliferation, molecular, immune, and chemotherapy-response parameters. TFAP2B-dominant tumors were associated with a coherent luminal phenotype, including apocrine/lobular enrichment, strong MUCL1 and AR expression, high CK18, low SOX10, reduced proliferation, frequent wild-type p53 patterns and retained RB1 expression, and low PD-L1 expression. FOXC1-dominant tumors showed contrasting basal-like features. These associations were independently supported at the transcriptional and genomic levels in the METABRIC TNBC cohort. FOXC1-dominant tumors also showed more frequent chemotherapy-induced downstaging than TFAP2B-dominant tumors. Together, we identify TFAP2B as a marker associated with luminal differentiation in TNBC and support combined TFAP2B/FOXC1 assessment as a practical framework for identifying clinically relevant TNBC differentiation states.

**Supplementary Information:**

The online version contains supplementary material available at https://doi.org/10.1038/s41598-026-68832-9.

## Introduction

Triple-negative breast cancer (TNBC) accounts for approximately 10–15% of all breast cancer cases and is defined by the absence of oestrogen receptor (ER), progesterone receptor (PR), and HER2 expression. It represents a molecularly diverse tumor entity characterized by aggressive clinical behavior and an unfavorable prognosis, which complicates diagnostic classification and therapeutic decision-making^1,2^.

Gene expression profiling by Lehmann et al. demonstrated that TNBC can be subdivided into distinct molecular subtypes, including basal-like 1 (BL1), basal-like 2 (BL2), mesenchymal (M), and luminal androgen receptor (LAR)^3,4^. Although these subtypes are acknowledged in the current WHO Classification of Tumors of the Breast, no reliable assay for routine subtype identification is currently available^5^. In this context, immunohistochemistry (IHC) may present a practical and widely applicable alternative for clinical implementation^6^.

Among these subtypes, LAR accounts for approximately 15% of TNBCs and is characterized by a luminal-like expression profile, molecular evidence of ER pathway activation, strong androgen receptor (AR) expression, and a close association with apocrine or lobular morphology^3,4,7^. Clinically, LAR tumors occur more frequently in older patients and are associated with lower proliferation rates and lower histological grade. Moreover, they respond poorly to neoadjuvant chemotherapy and are associated with low pathological complete response rates^3,8–10^. Nevertheless, published data regarding the prognostic significance of the LAR subtype remain inconsistent, with both favorable and adverse survival associations having been reported depending on the definition of LAR, including transcriptomic classification and AR-based surrogate approaches^11–14^.

Currently, AR represents the only established IHC marker for the LAR subtype; however, its diagnostic accuracy is limited. Several AR-based marker panels have been proposed, yet their specificity remains insufficient for reliable subtype identification, underscoring the need for more robust diagnostic approaches^6,13,15,16^.

Against this background, less established molecules such as MUCL1 (small breast epithelial mucin, SBEM) have gained attention. MUCL1 shows highly restricted expression in normal breast epithelium and breast cancer, with only limited expression in other tissues, and has been discussed both as a potential therapeutic target and as a predictive marker of chemoresistance^17,18^. In a previous study, we were the first to describe MUCL1 as an IHC marker associated with the LAR subtype. We demonstrated that MUCL1 expression strongly correlates with high AR and FOXA1 levels and is associated with a coherent clinicopathological phenotype aligned with LAR-associated features^19^.

While MUCL1 is strongly associated with a LAR-aligned phenotype, it does not directly capture the transcriptional regulatory programs underlying LAR lineage identity. In our previous study, MUCL1 mRNA expression showed a strong correlation with the transcription factor TFAP2B, suggesting a potential link between MUCL1 expression and TFAP2B-associated transcriptional programs. We therefore focused on TFAP2B as a candidate lineage-associated transcription factor in LAR tumors, aiming to complement MUCL1 with a mechanistically informative marker.

TFAP2B (AP-2β), a member of the AP-2 family of transcription factors, exerts both activating and repressive functions and has been associated with AR expression, low Ki-67 indices, lower tumor grading, lobular morphology, and luminal differentiation in breast cancer^20,21^. However, its role in TNBC, including the LAR subtype, remains largely unexplored.

In contrast, FOXC1, a member of the forkhead box (FOX) family of transcription factors involved in the regulation of cell growth, survival, differentiation, and migration, has been associated with the basal-like phenotype^22^. In TNBC, FOXC1 expression has been linked to chemosensitivity and has been reported to correlate inversely with AR expression^23^.

Based on these considerations, we hypothesized that TFAP2B and FOXC1 mark opposing, luminal and basal differentiation states in TNBC. We therefore investigated whether combined immunohistochemical assessment of TFAP2B and FOXC1 can identify biologically coherent TNBC subgroups beyond conventional AR-based classification and MUCL1 expression alone. In addition, we assessed whether these lineage-associated markers are linked to distinct histological patterns, molecular and immune phenotypes, and response to neoadjuvant chemotherapy, thereby providing a practical framework for capturing clinically relevant differentiation states within TNBC.

## Material and methods

### Patients and tumor samples

The study cohort consisted of 105 female patients, diagnosed with triple-negative breast cancer. The histological subtypes included apocrine (n = 6), lobular (n = 6), medullary (n = 8), metaplastic cancer (n = 11), and breast cancer of no special type (n = 74). Formalin-fixed, paraffin-embedded (FFPE) breast cancer tissue samples were used for the determination of estrogen receptor (ER), progesterone receptor (PR), and HER2 status. Additionally, the Ki-67 proliferation index was evaluated. Clinico-pathological and demographic data were obtained from clinical databases, pathological reports, and the Bavarian Cancer Registry. Tumor histology and grading were classified according to the 2019 WHO Classification of Tumors of the Breast. All patients underwent curative surgery at the Interdisciplinary Breast Center of Klinikum rechts der Isar, Technical University of Munich (TUM). Treatment recommendations were formulated through case discussions at an interdisciplinary tumor board. Patients receiving neoadjuvant chemotherapy were treated predominantly with anthracycline-/taxane-based regimens, including epirubicin, cyclophosphamide, paclitaxel, and carboplatin. Minor variations in treatment regimens occurred according to individual clinical indications and treatment standards.

### TMA

In this study, a tissue microarray (TMA) was constructed using tumor samples with a core diameter of 2 mm, with each sample represented in triplicate. The TMA construction was performed using the TMA Grand Master device (3DHISTECH). Tumor regions were carefully annotated on both the histological slides and the corresponding tissue blocks by expert gynecopathologists (F.-L.K., A.M.) to ensure accurate sampling. Tumor samples described in the patient and tumor sample section were included in the TMA. Kidney tissue served as an internal control for spatial orientation and, where appropriate, as a positive staining control. The resulting TMAs were used for subsequent immunohistochemical analyses. During processing, 6 of 315 TMA cores (1.9%) were lost.

### Immunohistochemistry

Immunohistochemical staining was performed on TMA sections from archival breast cancer resection specimens. Antibodies and staining specifications, including clone, supplier, and dilution, are listed in the Supplementary Material.

All stainings were carried out using an automated immunostaining system (BenchMark XT, Ventana Medical Systems, Tucson, AZ, USA) with 3,3′-diaminobenzidine (DAB) as the chromogen and the ultraView detection system.

Ki-67 was evaluated according to the recommendations of the International Ki-67 in Breast Cancer Working Group. For AR and FOXA1, nuclear immunoreactivity was quantified using the H-score. For analyses requiring binary classification, AR positivity was defined as an H-score ≥ 1. For TFAP2B, FOXC1, MUCL1, GCDFP-15, SOX10, GATA3, and TRPS1, a product score was used, combining staining intensity (0 = negative, 1 = low, 2 = moderate, 3 = high) and the percentage of positive tumor cells (0 =  < 1%, 1 = 1–10%, 2 = 11–50%, 3 = 51–100%). For TFAP2B and FOXC1, tumors with a product score ≥ 2 were considered positive.

Normal breast tissue served as a positive control for TFAP2B staining and showed the expected nuclear immunoreactivity in a subpopulation of luminal mammary epithelial cells, with absence of staining in myoepithelial cells.

AURKA expression was assessed by estimating the percentage of positive tumor cells in hotspot areas. CK18 staining was evaluated using an intensity score from 0 to 3 (negative, low, intermediate, high). For RB1, nuclear staining was scored as follows: 0 =  < 1%, 1 = 1–10%, 2 = 11–50%, 3 =  ≥ 50%. p53 expression was evaluated using a pattern-based approach as previously described^24^. PD-L1 immune cells (IC) were scored using the SP142 assay as the percentage of tumor area occupied by PD-L1–positive tumor-infiltrating immune cells. For categorical analyses and visualization, cases were grouped into IC 0%, 1–5%, 5–10%, 10–20%, and > 20% PD-L1–positive immune cells.

For supplementary categorical analyses, TFAP2B and FOXC1 expression was classified as negative (score 0), low (scores 1–4) or high (scores 6 or 9). Based on TFAP2B and FOXC1 immunohistochemical expression, tumors were classified using a TFAP2B/FOXC1 dominance-based three-group scheme comprising TFAP2B-dominant, FOXC1-dominant, and double-negative tumors. In cases with concomitant TFAP2B and FOXC1 expression (product score ≥ 2 for both markers), dominance was assigned according to the higher semi-quantitative immunohistochemical score. Tumors lacking expression of both markers were classified as double-negative. For selected analyses, an expanded five-group TFAP2B/FOXC1 expression-based classification was applied, further subdividing double-positive tumors according to relative marker dominance. Immunohistochemical scoring was performed in a blinded manner using predefined criteria. All cases were subsequently reviewed by expert gynecopathologists, and discrepant or equivocal findings were jointly reassessed and resolved by consensus.

### Assessment of chemotherapy response

Chemotherapy response was assessed by comparing the clinical or imaging-based tumor stage prior to neoadjuvant therapy (cT or iT, as documented in pathology reports) with the post-treatment pathological tumor stage (ypT) in patients who received neoadjuvant chemotherapy.

Response categories were defined as downstaging (lower ypT compared with initial cT/iT), stable disease (identical cT/iT and ypT), or progressive disease (higher ypT after therapy).

### Statistical analysis

All statistical analyses were performed using R software (version 4.4.1).

Categorical clinicopathological variables (e.g., tumor stage, molecular or immunohistochemical subgroups) were compared between TFAP2B- and FOXC1-defined groups using Pearson’s chi-square test or Fisher’s exact test, as appropriate, depending on expected cell counts.

Continuous variables (e.g., AURKA, Ki-67, age) were assessed for normality and analyzed using either Student’s t-test or the Wilcoxon rank-sum test, as appropriate.

The Wilcoxon rank-sum test was also applied for comparisons of ordinal variables between two groups (e.g., GCDFP-15 scores). For comparisons across more than two groups (e.g., MUCL1-negative, -low, and -high), the Kruskal–Wallis test was used.

Multivariable Firth logistic regression was used to identify clinicopathological factors independently associated with TFAP2B-dominant versus FOXC1-dominant status. The model included age at diagnosis, histological grade, pathological T stage, and Ki-67. Separate Firth logistic regression models assessed the association between TFAP2B/FOXC1 dominance and tumor downstaging versus stable disease or progression, adjusting for pretreatment T stage and, additionally or alternatively, Ki-67 or histological grade. FOXC1-dominant tumors served as the reference group. Age and Ki-67 were entered as continuous variables per 10-year and 10-percentage-point increase, respectively.

For analyses involving multiple biomarkers, *p* values were adjusted for multiple testing using the Benjamini–Hochberg false discovery rate method within biologically related groups of markers.

All *p*-values were two-sided, and a *p* < 0.05 was considered statistically significant.

### METABRIC cohort analysis

Publicly available transcriptomic and genomic data from triple-negative breast cancer cases in the METABRIC cohort^25^ were used for external validation. TFAP2B and FOXC1 mRNA expression levels were analyzed using Spearman rank correlations, and a continuous FOXC1–TFAP2B alignment score was calculated by subtracting the standardized TFAP2B expression value from the standardized FOXC1 expression value. Standardized expression values were obtained by subtracting the cohort mean and dividing by the cohort standard deviation. Higher scores indicated relatively greater FOXC1 expression, whereas lower scores indicated relatively greater TFAP2B expression. Associations with clinicopathological variables and marker expression were assessed using Spearman correlation or Wilcoxon rank-sum tests, as appropriate. Univariable logistic regression was used to evaluate associations of the alignment score with TP53 mutation and RB1 deletion. *p* values for multiple marker correlations were adjusted using the Benjamini–Hochberg method.

### Use of artificial intelligence tools

ChatGPT (OpenAI, San Francisco, USA) was used to assist with language refinement of the manuscript only. All scientific content was written, verified, and approved by the authors.

## Results

### Combined TFAP2B/FOXC1 stratification reveals distinct clinicopathological profiles in TNBC

**Fig. 1:**
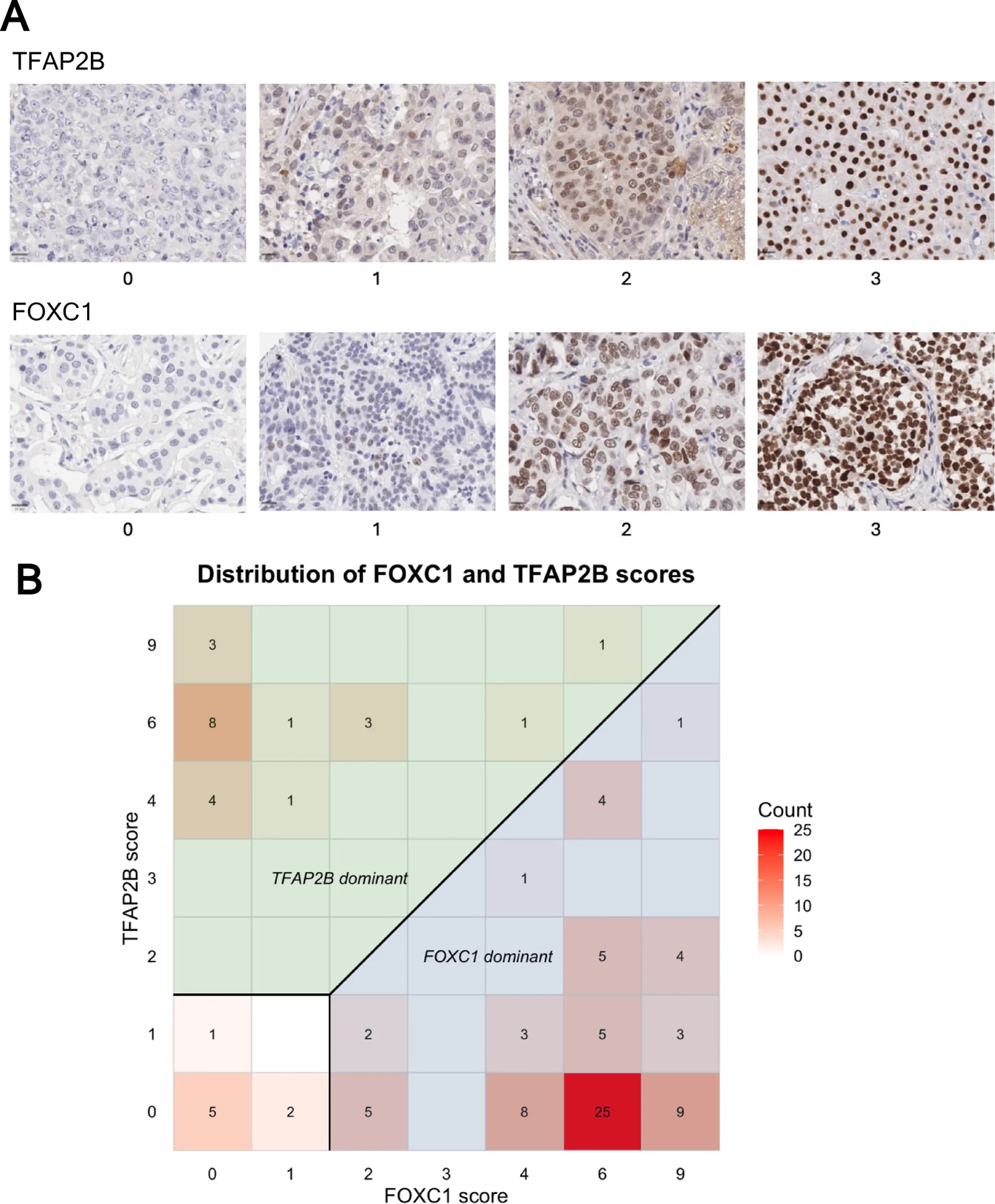
TFAP2B and FOXC1 expression patterns in TNBC. (**A**) Representative images illustrating four TFAP2B and FOXC1 nuclear staining intensity scores (0–3). Sections are counterstained with hematoxylin. Magnification 400×, scale bars 20 µm. (**B**) Heatmap showing the frequency of cases according to immunohistochemical FOXC1 (x-axis) and TFAP2B (y-axis) scores. The black lines indicate the predefined positivity cutoff (score ≥ 2). The diagonal line delineates relative dominance of FOXC1 versus TFAP2B expression among positive cases, separating FOXC1-dominant (blue-shaded area; FOXC1 > TFAP2B) from TFAP2B-dominant tumors (green-shaded area; TFAP2B > FOXC1).

Based on the hypothesized role of TFAP2B as a luminal epithelial differentiation marker in TNBC, immunohistochemical analysis demonstrated heterogeneous nuclear TFAP2B expression, ranging from complete absence to strong diffuse nuclear staining. FOXC1 also showed variable nuclear expression across the cohort (Fig. 1A). Although TFAP2B and FOXC1 expression patterns were largely distinct, among 105 tumors, 17 tumors showed exclusive TFAP2B expression, 60 showed exclusive FOXC1 expression, and 20 exhibited concomitant expression of both markers.

To enable an integrated and clinically interpretable assessment of TFAP2B- and FOXC1-associated tumor phenotypes, cases were grouped into three categories: double-negative tumors, TFAP2B-dominant tumors, and FOXC1-dominant tumors (Fig. 1B). Dominance was defined based on semi-quantitative immunohistochemical scores; in double-positive tumors (score ≥ 2 for both markers), classification was assigned according to the higher staining score (no cases with identical TFAP2B and FOXC1 scores were observed). Representative serial immunohistochemical stainings illustrating the contrasting immunophenotypes of TFAP2B- and FOXC1-dominant tumors are shown in the Supplementary Material.

Comparative clinicopathological analysis revealed marked differences across TFAP2B/FOXC1-defined subgroups (Table 1; *p* values refer to overall group comparisons). FOXC1-dominant tumors tended to occur in younger patients and were more frequently diagnosed as T1 tumors, whereas TFAP2B-dominant tumors were more often associated with advanced primary tumors (*p* = 0.0017 and *p* = 0.0004, respectively).

**Table 1:** Clinicopathological characteristics according to TFAP2B/FOXC1 expression status.

Variable	Double negative	TFAP2B dominant	FOXC1 dominant	*p*-value
n	n = 8	n = 22	n = 75	
Age at diagnosis	70.2 ± 14.2	66.4 ± 13.2	56.2 ± 14.5	0.0017
T2–4	8 (100%)	18 (81.8%)	35 (46.7%)	0.0004
T1		4 (18.2%)	40 (53.3%)	
N+	4 (57.1%)	12 (63.2%)	24 (34.8%)	0.0762
N0	3 (42.9%)	7 (36.8%)	45 (65.2%)	
G1/G2	4 (50%)	12 (54.5%)	11 (15.1%)	0.0004
G3	4 (50%)	10 (45.5%)	62 (84.9%)	
Ki-67 (MIB-1)	19.2 ± 24.3	10.5 ± 14.9	28.2 ± 25.3	0.0052
AURKA	7.5 ± 7.8	5.6 ± 6.9	14.2 ± 11.8	0.0009
Neoadjuvant CTx	4 (50%)	14 (63.6%)	45 (60.8%)	0.8460

Clinicopathological parameters are summarized for three TFAP2B/FOXC1-defined subgroups. Continuous variables are presented as mean ± standard deviation, and categorical variables as number (percentage). T status refers to tumor size category (T2-T4 vs. T1), N status indicates nodal involvement (N +), grading is grouped as G1/G2 versus G3, and proliferation is assessed by Ki-67 (MIB-1) and AURKA expression. *p* values indicate global comparisons across all three groups. Continuous variables were compared using the Kruskal–Wallis test, and categorical variables using Fisher’s exact test.

Histological grade differed significantly across subgroups (*p* = 0.0004), with high-grade carcinomas (G3) predominating in FOXC1-dominant tumors, while TFAP2B-dominant tumors were enriched for low- to intermediate-grade morphology. In line with this pattern, proliferative activity showed a clear gradient across the TFAP2B/FOXC1 axis. Ki-67 indices were lowest in TFAP2B-dominant tumors and highest in FOXC1-dominant tumors (*p* = 0.0052), with AURKA expression following a comparable distribution (*p* = 0.0009). In contrast, nodal status and the proportion of patients receiving neoadjuvant chemotherapy did not differ significantly across TFAP2B/FOXC1-defined subgroups. Compared with FOXC1-dominant tumors, double-negative cases were associated with older patient age and differed in T stage and histological grade. In contrast, no significant differences were observed with respect to proliferative markers.

Within TFAP2B-positive tumors, proliferative activity differed markedly according to concomitant FOXC1 expression: Ki-67 and AURKA levels were lowest in TFAP2B+/FOXC1− tumors and increased substantially in double-positive tumors, including TFAP2B-dominant TFAP2B+/FOXC1+ cases (Supplementary Table S1).

Multivariable Firth logistic regression confirmed that lower histological grade, higher pathological T stage, and lower Ki-67 were independently associated with TFAP2B dominance, whereas the association with older patient age did not remain significant after adjustment (Supplementary Table S2).

### TFAP2B/FOXC1 subgroups define histologic distribution and LAR-associated marker expression

**Fig. 2:**
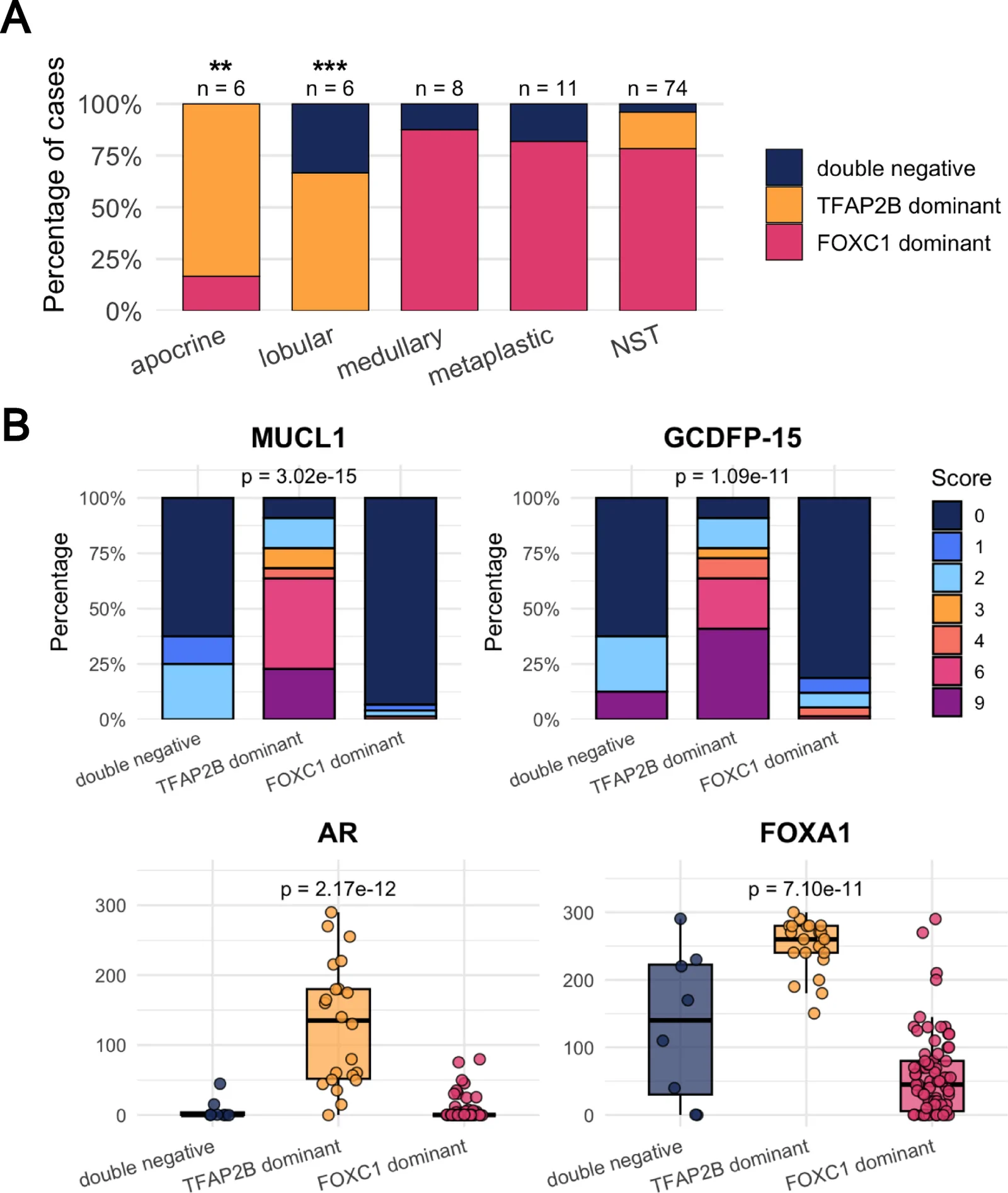
Histological distribution and LAR marker expression across TFAP2B/FOXC1-defined TNBC subgroups. (**A**) Relative frequencies of TFAP2B/FOXC1 expression groups across histological TNBC subtypes, shown as stacked percentages. Numbers above bars indicate sample size. Asterisks denote significant differences versus NST after Benjamini–Hochberg correction of Fisher’s exact tests. (**B**) Upper panels show the distribution of MUCL1 and GCDFP-15 immunohistochemical scores across TFAP2B/FOXC1 expression groups as stacked percentages. Lower panels display androgen receptor (AR) and FOXA1 H-scores as box-and-dot plots. Boxes represent the median and interquartile range; dots indicate individual cases. The overall subgroup sizes were n = 8 for double-negative tumors, n = 22 for TFAP2B-dominant tumors, and n = 75 for FOXC1-dominant tumors. *p* values were calculated using global Kruskal–Wallis tests and adjusted across the four displayed markers using the Benjamini–Hochberg false discovery rate procedure.

As shown in Fig. 2A, TFAP2B/FOXC1 expression patterns differed markedly across histologic subtypes. Apocrine carcinomas were strongly enriched for TFAP2B-dominant tumors (5/6, 83.3%), with only a minor fraction showing FOXC1 dominance (1/6, 16.7%). Similarly, lobular carcinomas were characterized by either TFAP2B-dominant (4/6, 66.7%) or double-negative phenotypes (2/6, 33.3%). In contrast, medullary and metaplastic carcinomas were predominantly FOXC1-dominant, accounting for 87.5% (7/8) and 81.8% (9/11) of cases, respectively, with the remaining cases in both subtypes classified as double-negative (1/8 and 2/11). NST carcinomas were likewise largely FOXC1-dominant (58/74, 78.4%), followed by TFAP2B-dominant (13/74, 17.6%) and double-negative tumors (3/74, 4.1%). Compared with NST carcinomas, the distribution of TFAP2B/FOXC1 dominance differed significantly for apocrine and lobular subtypes (*p* = 0.005 and *p* < 0.001, respectively).

Expression of LAR-associated markers differed markedly across TFAP2B/FOXC1-defined subgroups (Fig. 2B). MUCL1 expression showed a highly significant global association with TFAP2B/FOXC1 status (*p* = 3.0 × 10^−15^). TFAP2B-dominant tumors exhibited strong MUCL1 expression, with high-level staining (score ≥ 6) in 63.6% of cases, whereas MUCL1 expression was almost entirely absent in FOXC1-dominant tumors (score 0 in 93.3%). Double-negative tumors (n = 8) displayed heterogeneous MUCL1 expression, with the majority showing absent staining (62.5%), while a minority exhibited low-level expression.

A similarly pronounced pattern was observed for androgen receptor (AR) expression (*p* = 2.2 × 10^−12^). TFAP2B-dominant tumors showed high AR levels (median H-score 135, IQR 51.8–180), whereas AR expression was largely absent in FOXC1-dominant tumors (median H-score 0). Double-negative tumors again exhibited low and variable AR expression.

GCDFP-15 and FOXA1 expression followed the same overall trend, with enrichment in TFAP2B-dominant tumors and reduced expression in FOXC1-dominant tumors (both *p* < 10^−10^), although with greater overlap between subgroups. Collectively, FOXC1-dominant tumors consistently lacked expression of canonical LAR-associated markers, while TFAP2B-dominant tumors displayed a coherent LAR-aligned immunophenotype.

To further assess the relevance of AR expression within FOXC1-dominant tumors, FOXC1-dominant/AR+ and FOXC1-dominant/AR− cases were compared (Table 2). Clinicopathological features and biomarker expression profiles were largely comparable between both subgroups.

**Table 2:** Clinicopathological and biomarker characteristics stratified by TFAP2B- or FOXC1-dominant expression and AR status.

Variable	TFAP2B dom/AR +	FOXC1 dom/AR-	FOXC1 dom/AR +	*p*-value
n	n = 21	n = 57	n = 18	
Age at diagnosis	67.6 ± 12.2	56.8 ± 14.6	54.5 ± 14.5	0.0042
T+	17 (81%)	28 (49.1%)	7 (38.9%)	0.0163
T1	4 (19%)	29 (50.9%)	11 (61.1%)	
N+	11 (61.1%)	17 (30.9%)	7 (50%)	0.0507
N0	7 (38.9%)	38 (69.1%)	7 (50%)	
G1/G2	12 (57.1%)	8 (14.5%)	3 (16.7%)	0.0005
G3	9 (42.9%)	47 (85.5%)	15 (83.3%)	
Ki-67 (MIB-1)	8.9 ± 13	28.6 ± 26.9	26.7 ± 20	0.0025
AURKA	4.9 ± 6.2	14 ± 11.9	14.8 ± 12	0.0005
c-MET H-score	102.9 ± 70.5	143.1 ± 79.5	167.8 ± 73.3	0.0235
MUCL1 positive	20 (95.2%)	3 (5.3%)	2 (11.1%)	0.0001
SOX10 positive	0 (0%)	47 (82.5%)	15 (83.3%)	0.0001

Continuous variables are presented as mean ± SD; categorical variables as n (%). *p*-values indicate overall group comparisons. TFAP2B-dominant/AR − cases (n = 1) were excluded from statistical analyses.

When TFAP2B-dominant tumors were further stratified by FOXC1 expression (Fig. S1), additional heterogeneity became apparent within TFAP2B-positive tumors. Compared with TFAP2B+/FOXC1− cases, which uniformly expressed MUCL1, concomitant FOXC1 expression was associated with a marked reduction in MUCL1 expression, even when TFAP2B remained dominant. GCDFP-15 and AR expression showed a similar but less pronounced attenuation, whereas FOXA1 expression was largely preserved in TFAP2B-dominant tumors and declined primarily in FOXC1-dominant cases.

### Expression of basal and luminal lineage markers across TFAP2B/FOXC1-defined subgroups

**Fig. 3:**
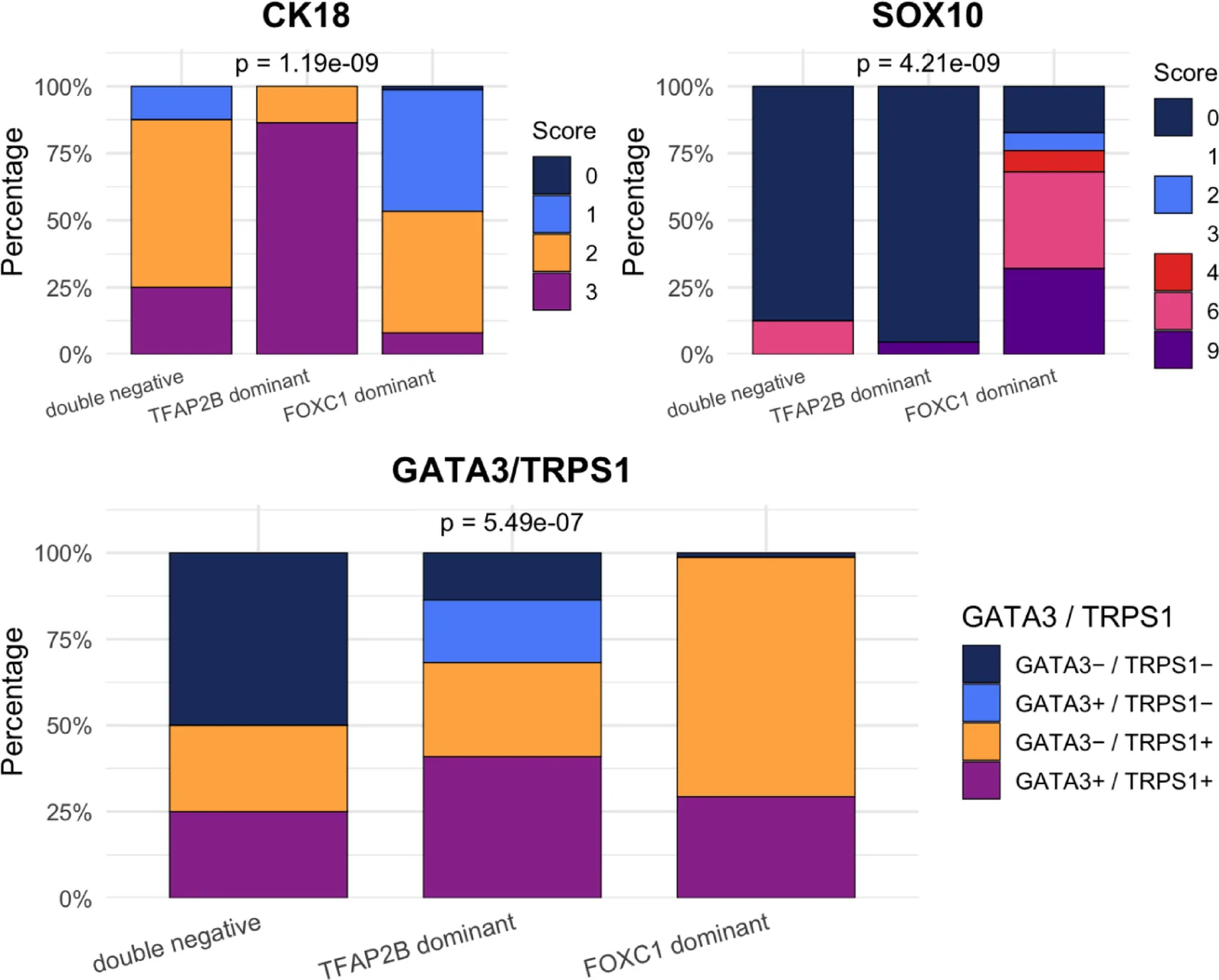
Differential expression of epithelial and basal markers across TFAP2B/FOXC1-defined TNBC subgroups. Stacked bar plots illustrate the percentage distribution of immunohistochemical staining scores for CK18 (upper left) and SOX10 (upper right) across the three TFAP2B/FOXC1 expression groups. CK18 scores range from 0 to 3, whereas SOX10 scores range from 0 to 9. The lower panel shows the proportional distribution of combined GATA3/TRPS1 expression patterns within the same groups, categorized as double negative, single positive, or double positive. The overall subgroup sizes were n = 8 for double-negative tumors, n = 22 for TFAP2B-dominant tumors, and n = 75 for FOXC1-dominant tumors. *p* values were calculated using global Kruskal–Wallis tests for CK18 and SOX10 and Fisher’s exact test for the GATA3/TRPS1 combinations, and were adjusted across the three comparisons using the Benjamini–Hochberg procedure.

Expression of additional lineage-associated markers differed significantly across TFAP2B/FOXC1-defined subgroups (Fig. 3). Cytokeratin 18 (CK18) intensity showed a strong global association with TFAP2B/FOXC1 status (*p* = 1.19 × 10^−9^). TFAP2B-dominant tumors exhibited predominantly strong CK18 expression, with high-intensity staining (score 3) observed in 86.4% of cases, consistent with a luminal epithelial phenotype. In contrast, FOXC1-dominant tumors demonstrated a marked shift towards lower CK18 intensities, with weak to moderate staining (scores 1–2) accounting for 90.6% of cases, while high-intensity CK18 expression was rare (8.0%). Double-negative tumors displayed an intermediate pattern with heterogeneous CK18 expression.

SOX10 expression also differed significantly across subgroups (*p* = 4.21 × 10^−9^). SOX10 was largely absent in TFAP2B-dominant tumors (95.5% score 0), whereas FOXC1-dominant tumors frequently expressed SOX10, with high scores (≥ 6) observed in 68.0% of cases. Double-negative tumors were predominantly SOX10-negative, with SOX10 expression largely confined to FOXC1-dominant tumors.

Combined analysis of GATA3 and TRPS1 further supported distinct luminal and basal differentiation states across TFAP2B/FOXC1-defined subgroups (Fisher’s exact test, *p* = 5.49 × 10^−7^). TFAP2B-dominant tumors were enriched for GATA3-positive phenotypes (59.1%), including both GATA3+/TRPS1− and GATA3+/TRPS1+ cases, whereas FOXC1-dominant tumors were characterized by a predominance of GATA3-negative/TRPS1-positive tumors (69.3%) and lacked GATA3-positive/TRPS1-negative cases. Double-negative tumors showed a mixed distribution across GATA3/TRPS1 categories.

### FOXC1-associated differences in p53 status, immune markers, and c-MET expression

**Fig. 4:**
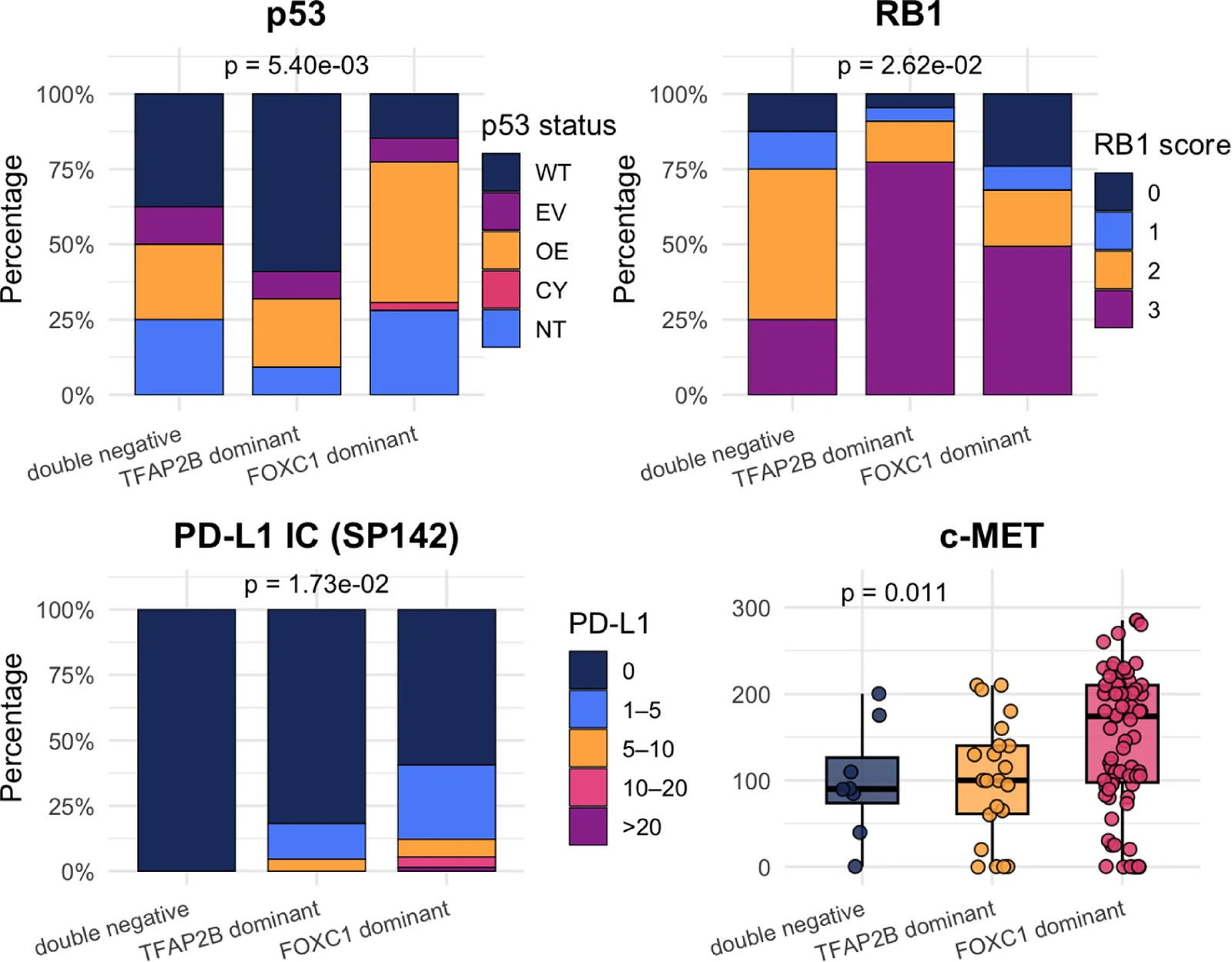
Association of p53 status, RB1 expression, PD-L1 IC (SP142), and c-MET with TFAP2B/FOXC1-defined subgroups in triple-negative breast cancer. Stacked bar plots illustrate the distribution of p53 immunohistochemical expression patterns (WT, wild-type; EV, equivocal; OE, overexpression; CY, cytoplasmic; NT, null-type; upper left) and RB1 expression scores (0–3; upper right) across the three TFAP2B/FOXC1 subgroups. The lower left panel shows PD-L1 immune-cell staining (SP142) categorized as 0, 1–5, 5–10, 10–20, and > 20. c-MET expression is displayed as box-and-dot plots (H-score) with individual cases overlaid (lower right). Bars represent relative proportions (percentages) within each subgroup. The overall subgroup sizes were n = 8 for double-negative tumors, n = 22 for TFAP2B-dominant tumors, and n = 75 for FOXC1-dominant tumors. *p*-values indicate global group comparisons using Fisher’s exact test for p53 status and Kruskal–Wallis tests for RB1, PD-L1 IC, and c-MET.

As shown in Fig. 4, alterations in p53 status, RB1 expression, PD-L1 IC, and c-MET further distinguished molecular profiles across TFAP2B/FOXC1-defined subgroups.

p53 expression patterns differed significantly between groups (*p* = 5.40 × 10^−3^). TFAP2B-dominant tumors most frequently exhibited a wild-type p53 pattern (59.1%), whereas FOXC1-dominant tumors were enriched for aberrant p53 expression, with p53 overexpression and null-type patterns accounting for 46.7% and 28.0% of cases, respectively. Double-negative tumors showed an intermediate and heterogeneous distribution, with wild-type p53 detected in 37.5% of cases.

RB1 expression also differed across subgroups (*p* = 2.62 × 10^−2^). Strong RB1 expression (score 3) predominated in TFAP2B-dominant tumors (77.3%), whereas FOXC1-dominant tumors showed a relative shift towards lower RB1 scores, including complete RB1 loss in 24.0% of cases. Double-negative tumors displayed an intermediate pattern, with RB1 loss observed in 12.5%.

Analysis of PD-L1 IC (SP142) revealed significant differences between groups (*p* = 1.73 × 10^−2^). FOXC1-negative tumors were largely PD-L1–negative (double-negative: 100%; TFAP2B-dominant: 81.8%), whereas FOXC1-dominant tumors more frequently exhibited PD-L1 IC positivity, with 40.5% of cases showing PD-L1 expression ≥ 1%.

Finally, c-MET expression differed significantly across subgroups (*p* = 0.011). FOXC1-dominant tumors demonstrated markedly higher c-MET H-scores (median 174, IQR 97.5–210.0) compared with TFAP2B-dominant (median 100.0, IQR 61.2–140.0) and double-negative tumors (median 90.0, IQR 73.8–126.2).

When TFAP2B-dominant tumors were further stratified by FOXC1 expression (Fig. S2), additional heterogeneity within TFAP2B-positive tumors became apparent. In particular, TFAP2B+/FOXC1− tumors retained a luminal profile, with predominantly wild-type p53 patterns (70.6%), low PD-L1 expression (5.9%), and lower c-MET levels. In contrast, acquisition of FOXC1 expression was associated with aberrant p53 patterns, increased PD-L1 expression, and higher c-MET expression, even in TFAP2B-dominant cases.

### Chemotherapy response stratified by TFAP2B/FOXC1 status

**Fig. 5:**
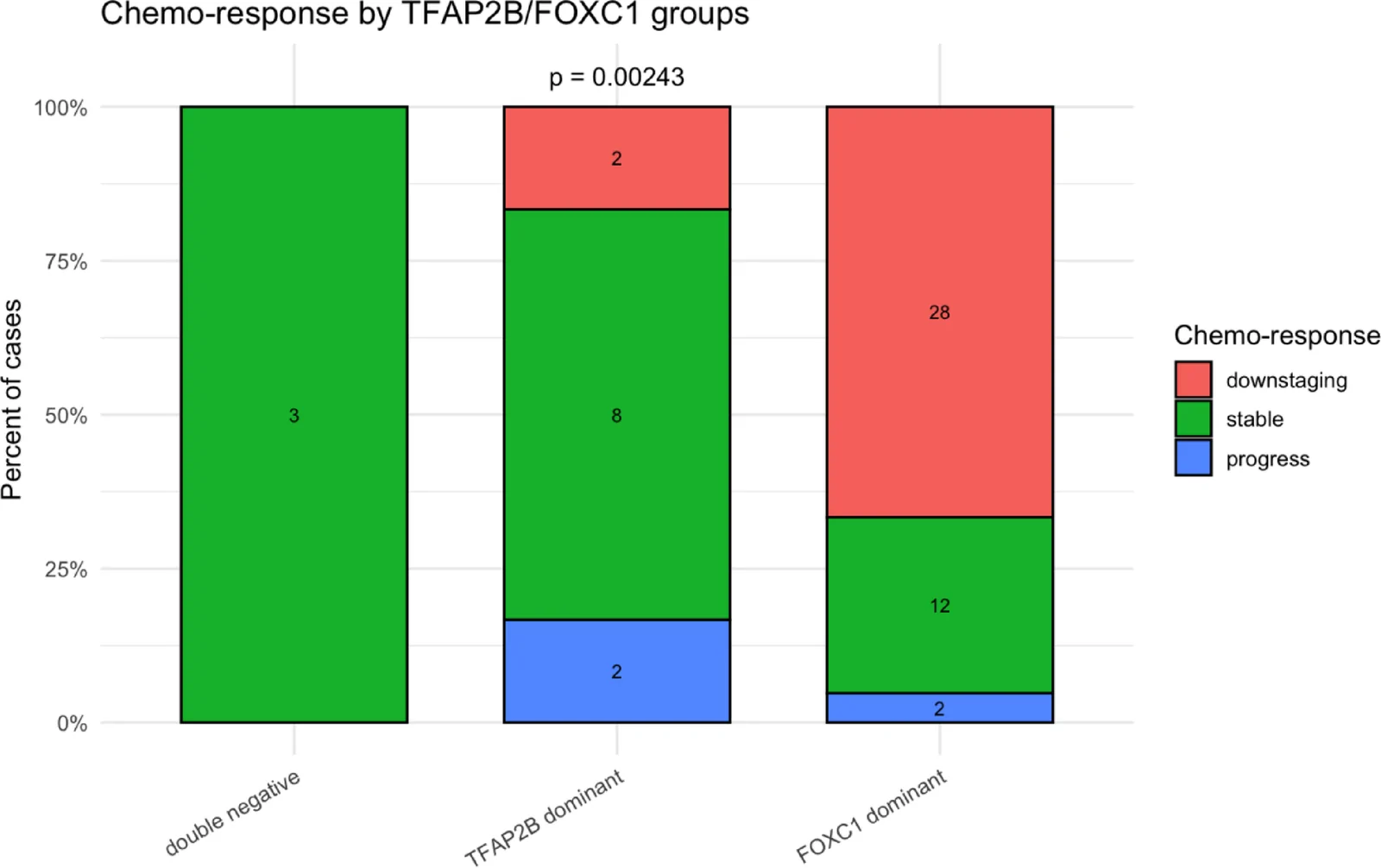
Chemotherapy-response according to combined TFAP2B/FOXC1 expression status. Stacked bar plots show the relative distribution of neoadjuvant chemo-response categories (downstaging, stable disease, progression) across the three TFAP2B/FOXC1 groups. Numbers within bars indicate absolute case counts; bar heights represent percentages within each group. The overall association between TFAP2B/FOXC1 group and chemo-response was assessed using Fisher’s exact test.

Chemotherapy response differed significantly across TFAP2B/FOXC1-defined subgroups (*p* = 0.0024; Fig. 5). Owing to the very small size of the TFAP2B−/FOXC1− group with neoadjuvant chemotherapy (n = 3; all stable disease), comparative analyses focused on TFAP2B- and FOXC1-dominant tumors.

TFAP2B-dominant tumors showed a substantially lower rate of downstaging, with responses observed in only 16.7% (2/12) of cases, while progressive disease occurred at a comparable frequency (16.7%, 2/12). In contrast, FOXC1-dominant tumors demonstrated a markedly higher rate of downstaging (66.7%, 28/42), whereas progression was rare (4.8%, 2/42). Stable disease accounted for the remaining cases in both groups.

Consistent with this combined-group analysis, single-marker evaluation showed an association between FOXC1 expression and treatment response (Fig. S3): tumors with high FOXC1 expression exhibited downstaging in 62.5% (20/32) of cases, compared with 18.2% (2/11) in FOXC1-negative tumors, with a trend toward statistical significance (*p* = 0.062). In contrast, TFAP2B status alone showed an inverse association with chemosensitivity, as TFAP2B-high tumors rarely downstaged (9.1%, 1/11) and more frequently displayed stable or progressive disease, whereas TFAP2B-negative and TFAP2B-low tumors showed substantially higher downstaging rates (58.6% and 70.5%, respectively; *p* = 0.001).

Multivariable Firth logistic regression showed that TFAP2B-dominant tumors remained significantly less likely to undergo tumor downstaging than FOXC1-dominant tumors after accounting for pretreatment T stage (OR 0.14, 95% CI 0.02–0.58, *p* = 0.006). This association remained robust in additional multivariable models including Ki-67 (OR 0.12, *p* = 0.005) or histological grade (OR 0.16, *p* = 0.015; Supplementary Table S3).

### Transcriptional and genomic validation in the METABRIC cohort

**Fig. 6:**
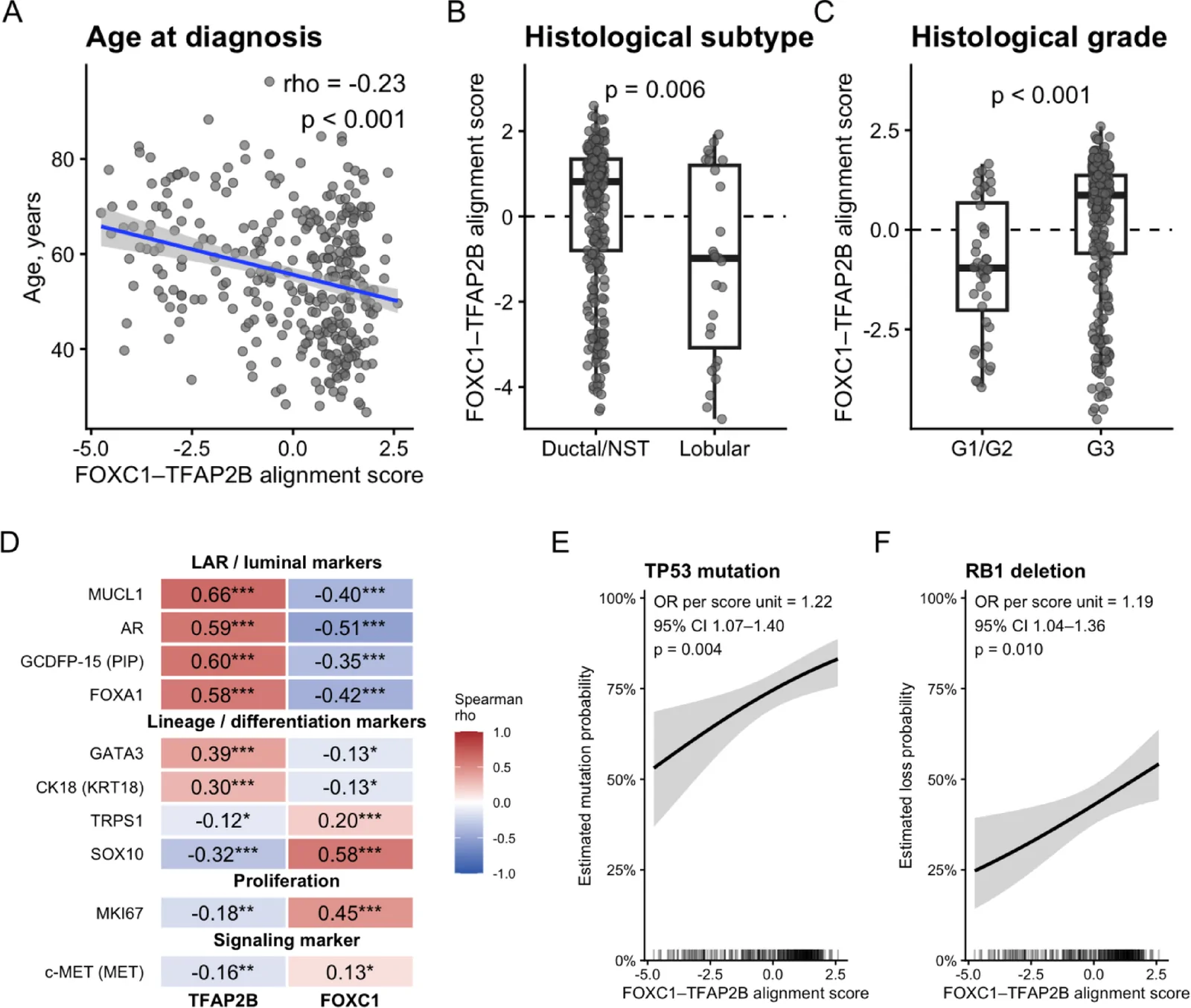
Transcriptional and genomic validation of the TFAP2B–FOXC1 axis in triple-negative breast cancer cases from the METABRIC cohort. (**A**) Association of the continuous FOXC1–TFAP2B alignment score with age at diagnosis. The line indicates the fitted relationship; the association was assessed using Spearman rank correlation. (**B**) Distribution of the alignment score according to histological subtype (ductal/NST versus lobular). (**C**) Distribution of the alignment score according to histological grade (G1/G2 versus G3). (**D**) Spearman correlations of TFAP2B and FOXC1 expression with selected luminal, lineage/differentiation, proliferation, and signaling-associated markers. Cell values indicate Spearman’s ρ; asterisks denote Benjamini–Hochberg-adjusted significance levels (*p < 0.05; **p < 0.01; ***p < 0.001). (**E**, **F**) Predicted probabilities of TP53 mutation and RB1 deletion according to the continuous FOXC1–TFAP2B transcriptional alignment score, calculated as z(FOXC1) − z(TFAP2B). Probability estimates and 95% confidence intervals were derived from univariable logistic regression models. Odds ratios are reported per one-unit increase in the alignment score. The dashed vertical line marks an alignment score of zero. RB1 deletion was defined as a discrete copy-number value ≤  − 1.

To validate the TFAP2B–FOXC1 axis at the transcriptional level, we analyzed triple-negative breast cancer cases from the METABRIC cohort. TFAP2B and FOXC1 expression were inversely correlated (Spearman ρ =  − 0.37, *p* < 0.001; n = 320; Fig. S4). We then defined a continuous alignment score as z(FOXC1) − z(TFAP2B), with higher values indicating greater FOXC1 alignment. Higher scores were weakly associated with younger age at diagnosis (Spearman ρ =  − 0.23, *p* < 0.001; Fig. 6A). Scores were also higher in ductal/NST than in lobular carcinomas (Wilcoxon *p* = 0.006; Fig. 6B) and in G3 than in G1/G2 tumors (Wilcoxon *p* < 0.001; Fig. 6C).

TFAP2B showed strong positive correlations with luminal-associated markers, including MUCL1, AR, PIP/GCDFP-15, and FOXA1 (ρ = 0.58–0.66), whereas FOXC1 was inversely correlated with these markers (ρ =  − 0.35 to − 0.51; all BH-adjusted *p* < 0.001; Fig. 6D). FOXC1 correlated positively with SOX10 and MKI67, while TFAP2B correlated inversely with both. GATA3 and KRT18 were positively associated with TFAP2B and weakly inversely associated with FOXC1. TRPS1 and MET showed weak inverse associations with TFAP2B and positive associations with FOXC1.

In univariable logistic regression analyses, increasing alignment scores were associated with higher probabilities of TP53 mutation (OR per score unit = 1.22, 95% CI 1.07–1.40, *p* = 0.004; Fig. 6E) and RB1 deletion (OR per score unit = 1.19, 95% CI 1.04–1.36, *p* = 0.010; Fig. 6F).

## Discussion

Gene expression profiling has identified multiple molecular subtypes of triple-negative breast cancer (TNBC), including basal-like (BL1/BL2), mesenchymal, and luminal androgen receptor (LAR) subtypes^4^. While these subtypes differ in their transcriptional programs and clinical behavior, subtype classification relies predominantly on mRNA expression data, and the extent to which epithelial lineage-associated differentiation programs are reflected at the protein level remains insufficiently characterized. In this context, we investigated FOXC1 and TFAP2B as immunohistochemical markers of basal and luminal differentiation states in TNBC.

Consistent with its established association with the basal-like subtype, high FOXC1 expression has been reported at both mRNA and protein levels in basal-like TNBCs, reflecting a basal epithelial differentiation program^16,26^. Clinically, FOXC1-positive TNBCs are characterized by an aggressive phenotype and poor survival^27^. This was recapitulated in our cohort, where FOXC1-dominant tumors exhibited high proliferative activity and high histological grade. FOXC1 expression is strongly associated with basal lineage markers, particularly SOX10^28,29^, and is typically accompanied by absence of androgen receptor (AR) expression^23^, underscoring its role as a marker of the basal-like differentiation axis. Within FOXC1-dominant tumors in our cohort, AR expression did not identify a distinct clinicopathological subset, suggesting that FOXC1-associated basal-like biology predominates over AR status in this context.

The transcription factor TFAP2B (AP-2β) is a marker of mammary epithelial differentiation that defines a luminal, CK18-positive epithelial subpopulation^21^. In hormone receptor-positive breast cancer, TFAP2B expression is enriched in lobular carcinomas and associated with lower histological grade, reduced proliferative activity, and AR positivity^20^. Accordingly, our data demonstrate that TFAP2B expression in TNBC is associated with luminal features, including enrichment of LAR-associated markers, almost complete absence of SOX10, and higher patient age, a constellation characteristic of the luminal androgen receptor (LAR) subtype of TNBC^9^. Despite lower proliferative activity, TFAP2B-dominant tumors presented with larger tumor size and a trend towards more frequent nodal involvement, possibly reflecting differences in clinical presentation or detectability between luminal- and basal-like TNBC phenotypes. Multivariable analysis confirmed independent associations of TFAP2B/FOXC1 dominance with histological grade, pathological T stage, and Ki-67, whereas the association with age did not remain significant after adjustment. Raap et al.^20^ also reported enrichment of TFAP2B expression in apocrine metaplasia, in line with our observation of increased expression in apocrine carcinomas. Together, these findings identify TFAP2B as a previously underexplored marker associated with luminal differentiation in TNBC and indicate that TFAP2B and FOXC1 mark opposing luminal and basal phenotypes at the protein level.

To further characterize TFAP2B/FOXC1-defined subgroups in TNBC, we examined TRPS1 expression. TRPS1 has been reported as a highly sensitive marker of mammary differentiation across a broad range of breast carcinomas, including TNBC, where it outperforms established markers such as GATA3 in non-luminal subtypes^30^. In contrast, TRPS1 expression is less frequent in apocrine, AR-positive or LAR-type TNBC^6,31,32^. Consistent with these observations, our data demonstrate that TRPS1 expression was significantly more frequent in FOXC1-dominant TNBC compared with TFAP2B-positive tumors. This supports its utility as an immunohistochemical marker in basal-like TNBC, while indicating reduced applicability in LAR TNBC. GATA3 expression has been reported to be more sensitive than TRPS1 in apocrine TNBCs^32^, a trend that was likewise observed in our cohort among TFAP2B-positive tumors.

Beyond differentiation-associated marker expression, our findings indicate that FOXC1 positivity is associated with more frequent aberrant p53 staining patterns and loss of RB1 protein expression, whereas TFAP2B-dominant tumors more often retain wild-type p53 patterns and intact RB1 expression. Aberrant p53 staining may serve as a surrogate for TP53 mutation status^24^, whereas loss of RB1 protein expression may reflect RB pathway dysfunction but does not directly establish an underlying RB1 genomic alteration. Basal breast cancers, which largely overlap with TNBC, harbor TP53 mutations in approximately 80% of cases and combined TP53/RB1 alterations in around 40%, substantially more often than luminal, hormone receptor-positive tumors^33–35^. A similar basal–luminal dichotomy has been described across TNBC molecular subtypes^36,37^, consistent with the enrichment of aberrant p53 and RB1 loss in FOXC1-dominant tumors and the more LAR-like profile of TFAP2B-dominant tumors.

The immune microenvironment differs substantially between basal and luminal breast cancers. PD-L1 expression is more common in basal tumors and reflects the presence of an immune-enriched tumor microenvironment associated with increased chemosensitivity and more favorable clinical outcomes in TNBC^38–40^. A similar pattern is observed within TNBC at the level of molecular subtypes, where basal-like tumors contrast with LAR tumors^36,41,42^. PD-L1 expression in immune cells was largely absent in TFAP2B-positive/FOXC1-negative tumors but enriched in FOXC1-expressing tumors, irrespective of TFAP2B status.

MET has been reported to be overexpressed in basal-like breast cancers and associated with poor survival^43^, and has therefore been discussed as a potential therapeutic target in subsets of TNBC^44^. Consistent with this, FOXC1-dominant tumors in our cohort exhibited significantly higher c-MET expression than TFAP2B-dominant tumors.

Importantly, the broader pattern of associations observed in our cohort, including those involving age, histological subtype and grade, luminal and basal lineage markers, proliferation, MET, TP53, and RB1, was independently supported in the METABRIC TNBC cohort^25^, providing external validation and strengthening the overall robustness of our findings.

Chemotherapy response represents a key dimension of heterogeneity within TNBC, with basal-like tumors showing higher response rates, whereas the LAR subtype is characterized by pronounced chemoresistance^4,8,45^. Consistent with this pattern, FOXC1-dominant tumors in our cohort exhibited higher rates of downstaging and low frequencies of progressive disease after neoadjuvant chemotherapy, while TFAP2B-dominant tumors were characterized by poor response and frequent stable or progressive disease. The association between TFAP2B/FOXC1 dominance and tumor downstaging remained significant in multivariable models adjusting for pretreatment T stage and, additionally or alternatively, Ki-67 or histological grade.

These findings indicate that TFAP2B- and FOXC1-associated phenotypes are linked to clinically relevant differences in chemosensitivity within TNBC. Beyond treatment response, these observations further support their association with broader differentiation states rather than isolated marker expression.

A more granular five-group TFAP2B/FOXC1 analysis, as presented in the supplementary data, identified intermediate phenotypes between luminal- and basal-associated TNBC. FOXC1-positive tumors lacking TFAP2B expression largely resembled double-positive tumors in which FOXC1 remained dominant, indicating that FOXC1 predominance is associated with a basal-like phenotype even in the presence of TFAP2B expression. Conversely, within TFAP2B-dominant tumors, concomitant FOXC1 expression was associated with a partial shift towards a more basal phenotype, including higher proliferation, reduced MUCL1 expression, more frequent aberrant p53 patterns, increased PD-L1 expression, and elevated c-MET levels. Although double-positive tumors constituted only a small fraction of the cohort, these findings indicate overlapping rather than discrete luminal- and basal-associated phenotypes. As a lineage-associated transcription factor, TFAP2B may therefore reflect luminal differentiation states beyond hormone receptor activity and complement downstream markers such as AR, MUCL1, or GCDFP15.

While the findings of the study support the proposed biological model, several limitations should be considered. Its retrospective single-center design and the limited size of some subgroups, particularly the double-positive tumors, restrict statistical power and generalizability. In addition, no functional experiments were performed, and the observed associations therefore do not establish a causal role of TFAP2B or FOXC1 in luminal or basal differentiation. Chemotherapy response was assessed using stage downstaging rather than pCR or RCB because the resection-based design systematically excluded pCR cases and complete RCB data were unavailable. Consequently, treatment-response analyses should be considered exploratory, and prognostic analyses require validation in independent pretreatment biopsy cohorts.

Together, these findings support a model in which FOXC1 and TFAP2B are associated with opposing yet not mutually exclusive, basal and luminal differentiation states in TNBC, accompanied by distinct molecular, immunophenotypic, and clinical features. Combined TFAP2B and FOXC1 assessment may therefore provide a practical immunohistochemical framework for identifying clinically relevant tumor phenotypes across the spectrum of luminal and basal differentiation states.

## Supplementary Information

Below is the link to the electronic supplementary material.

Supplementary Material 1

Supplementary Material 2

Supplementary Material 3

Supplementary Material 4

## Data Availability

The datasets generated during and/or analyzed during the current study are available from the corresponding author on reasonable request.
