# Supplementary material for: TFAP2B and FOXC1 are associated with biologically and clinically distinct differentiation states in triple-negative breast cancer
Source: Sci Rep. 2026 Sep 11;16:28458. doi: 10.1038/s41598-026-68832-9 (PMC13569491; doi:10.1038/s41598-026-68832-9)
Supplement: Supplementary file 1 [file 41598_2026_68832_MOESM2_ESM.pdf]

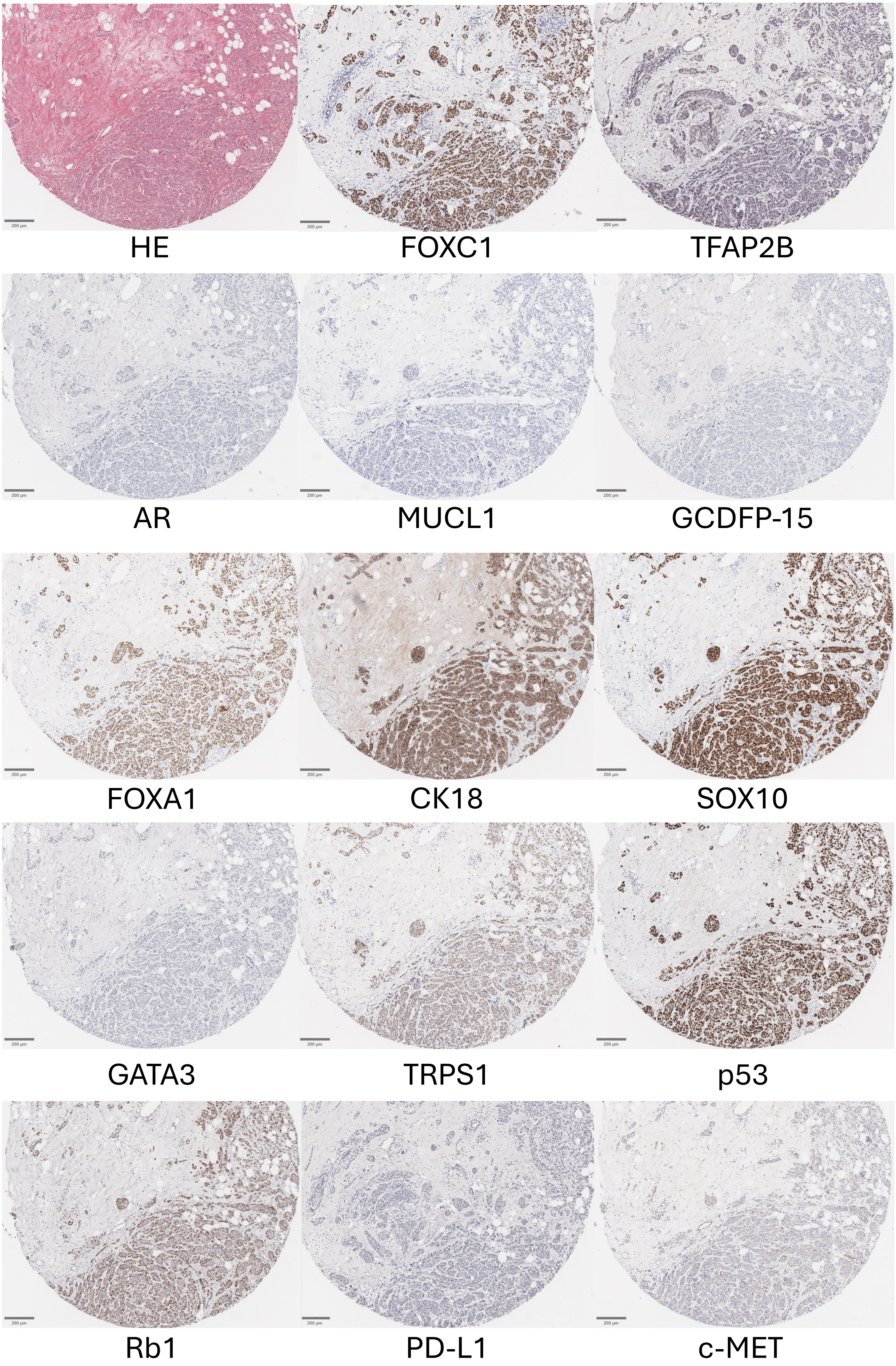
HE
FOXC1
TFAP2B
AR
MUCL1
GCDFP-15
FOXA1
CK18
SOX10
GATA3
TRPS1
p53
Rb1
PD-L1
c-MET

Supplementary Figure S5. Representative serial immunohistochemical stainings of a FOXC1-dominant triple-negative breast carcinoma.

Representative serial sections illustrating the immunophenotypic profile of a FOXC1-dominant tumor. The individual immunohistochemical markers are indicated in the figure.
