# Supplementary material for: TFAP2B and FOXC1 are associated with biologically and clinically distinct differentiation states in triple-negative breast cancer
Source: Sci Rep. 2026 Sep 11;16:28458. doi: 10.1038/s41598-026-68832-9 (PMC13569491; doi:10.1038/s41598-026-68832-9)
Supplement: Supplementary file 3 [file 41598_2026_68832_MOESM4_ESM.docx]

**Supplementary Material:**

| **Variable** | **TFAP2B-/FOXC1-** | **TFAP2B+/FOXC1-** | **TFAP2B-/FOXC1+** | **TFAP2B+/FOXC1+ (TFAP2B > FOXC1)** | **TFAP2B+/FOXC1+ (FOXC1 > TFAP2B)** | **p-value** | |
| --- | --- | --- | --- | --- | --- | --- | --- |
| n | n = 8 | n = 17 | n = 60 | n = 5 | n = 15 |  |  |
| Age at diagnosis | 70.7 ± 14.1 | 67.9 ± 13.5 | 57 ± 14.6 | 63.7 ± 13.2 | 55.5 ± 14.3 | 0.0107 |  |
| T+ | 8 (100%) | 14 (82.4%) | 28 (46.7%) | 4 (80%) | 7 (46.7%) | 0.0024 |  |
| T1 |  | 3 (17.6%) | 32 (53.3%) | 1 (20%) | 8 (53.3%) |  |  |
| N+ | 4 (57.1%) | 8 (57.1%) | 22 (40%) | 4 (80%) | 2 (14.3%) | 0.0455 |  |
| N0 | 3 (42.9%) | 6 (42.9%) | 33 (60%) | 1 (20%) | 12 (85.7%) |  |  |
| G1/G2 | 4 (50%) | 10 (58.8%) | 9 (15.5%) | 2 (40%) | 2 (13.3%) | 0.0014 |  |
| G3 | 4 (50%) | 7 (41.2%) | 49 (84.5%) | 3 (60%) | 13 (86.7%) |  |  |
| Ki-67 (MIB-1) | 19.2 ± 24.3 | 5.2 ± 6.9 | 26.2 ± 25 | 28.4 ± 21.3 | 35.8 ± 25.8 | 0.0012 |  |
| AURKA | 7.5 ± 7.8 | 3.9 ± 5.8 | 13.7 ± 12 | 11.2 ± 8 | 16.1 ± 11.5 | 0.0009 |  |
| Neo-adjuvant Ctx | 4 (50%) | 10 (58.8%) | 36 (61%) | 4 (80%) | 9 (60%) | 0.9123 |  |

**Table S1: Clinicopathological parameters according to expanded five-group TFAP2b/FOXC1-based classification.** Tumors were stratified based on TFAP2B and FOXC1 immunohistochemical expression, with double-positive cases further subdivided according to relative marker dominance (TFAP2B > FOXC1 vs. FOXC1 > TFAP2B). p-values indicate overall group comparisons.

| **model** | **n** | **predictor** | **OR (95% CI)** | **p-value** |
| --- | --- | --- | --- | --- |
| Univariable | 97 | Age at diagnosis, per 10 years | 1.66 (1.17–2.47) | 0.004 |
| Univariable | 95 | G3 vs G1/G2 | 0.15 (0.05–0.43) | <0.001 |
| Univariable | 97 | Pathological T2–4 vs T1 | 4.69 (1.64–16.23) | 0.003 |
| Univariable | 97 | Ki-67, per 10 percentage points | 0.64 (0.44–0.86) | 0.001 |
| Multivariable | 95 | Age at diagnosis, per 10 years | 1.32 (0.86–2.09) | 0.207 |
| Multivariable | 95 | G3 vs G1/G2 | 0.27 (0.08–0.86) | 0.027 |
| Multivariable | 95 | Pathological T2–4 vs T1 | 6.67 (1.95–28.33) | 0.002 |
| Multivariable | 95 | Ki-67, per 10 percentage points | 0.62 (0.39–0.89) | 0.006 |

**Supplementary Table S2. Clinicopathological factors associated with TFAP2B-dominant versus FOXC1-dominant tumors.** Univariable and multivariable Firth logistic regression analyses were performed using TFAP2B dominance as the binary outcome and FOXC1-dominant tumors as the reference category. The multivariable model included age at diagnosis, histological grade, pathological T stage, and Ki-67. Age was entered per 10-year increase and Ki-67 per 10-percentage-point increase. Odds ratios are shown with 95% confidence intervals.

**Figure S1. Association of expanded five-group TFAP2b/FOXC1-based expression groups with LAR marker expression in TNBC.**

Upper panels show the distribution of MUCL1 and GCDFP-15 immunohistochemical scores across TFAP2B/FOXC1 expression groups, displayed as stacked percentages. Lower panels depict androgen receptor (AR) and FOXA1 H-scores as box-and-dot plots. Boxes indicate median and interquartile range; dots represent individual tumors. TFAP2B+/FOXC1+ cases were further subdivided according to relative marker dominance (TFAP2B > FOXC1 vs. FOXC1 > TFAP2B). P values were calculated using the Kruskal–Wallis test.

**Figure S2. Association of p53 status, RB1 expression, PD-L1 IC (SP142), and c-MET with expanded five-group TFAP2b/FOXC1-based subgroups in triple-negative breast cancer.**

Stacked bar plots illustrate the distribution of p53 immunohistochemical expression patterns (WT, wild-type; EV, equivocal; OE, overexpression; CY, cytoplasmic; NT, null-type; upper left) and RB1 expression scores (0–3; upper right) across the five TFAP2B/FOXC1 subgroups. The lower left panel shows PD-L1 immune-cell staining (SP142) categorized as 0, 1–5, 5–10, 10–20, and >20. c-MET expression is displayed as box-and-dot plots (H-score) with individual cases overlaid (lower right).
Bars represent relative proportions (percentages) within each subgroup. P-values indicate global group comparisons using Fisher’s exact test for p53 status and Kruskal–Wallis tests for RB1, PD-L1 IC, and c-MET.

**Figure S3. Chemotherapy-response according to TFAP2B and FOXC1 expression status.**
Stacked bar plots illustrate the distribution of neoadjuvant chemo-response categories (downstaging, stable disease, progression) stratified by TFAP2B (left) and FOXC1 (right) expression levels (negative, low, high). Numbers within bars indicate absolute case counts; bar heights represent percentages within each expression category. Associations between marker expression status and chemo-response were evaluated using Fisher’s exact test.

| **model** | **n** | **predictor** | **OR (95% CI)** | **p-value** |
| --- | --- | --- | --- | --- |
| Model 0: dominance | 54 | TFAP2B dominant vs FOXC1 dominant | 0.12 (0.02–0.49) | 0.002 |
| Model 1: dominance + pretreatment T stage | 54 | TFAP2B dominant vs FOXC1 dominant | 0.14 (0.02–0.58) | 0.006 |
| Model 1: dominance + pretreatment T stage | 54 | Pretreatment T2–4 vs T1 | 5.12 (1.10–31.92) | 0.038 |
| Model 2: dominance + pretreatment T stage + Ki-67 | 54 | TFAP2B dominant vs FOXC1 dominant | 0.12 (0.02–0.55) | 0.005 |
| Model 2: dominance + pretreatment T stage + Ki-67 | 54 | Pretreatment T2–4 vs T1 | 5.66 (1.17–36.74) | 0.031 |
| Model 2: dominance + pretreatment T stage + Ki-67 | 54 | Ki-67, per 10 percentage points | 0.91 (0.69–1.19) | 0.480 |
| Model 3: dominance + pretreatment T stage + grade | 53 | TFAP2B dominant vs FOXC1 dominant | 0.16 (0.02–0.71) | 0.015 |
| Model 3: dominance + pretreatment T stage + grade | 53 | Pretreatment T2–4 vs T1 | 4.71 (1.02–28.87) | 0.047 |
| Model 3: dominance + pretreatment T stage + grade | 53 | G3 vs G1/G2 | 1.13 (0.26–4.55) | 0.860 |

**Supplementary Table S3. Firth logistic regression analyses of tumor downstaging according to TFAP2B/FOXC1 dominance.**

The binary outcome was tumor downstaging versus no downstaging, defined as stable disease or progression. FOXC1-dominant tumors served as the reference group. Model 0 was unadjusted; Model 1 was adjusted for pretreatment T stage; Model 2 was additionally adjusted for Ki-67; and Model 3 was alternatively adjusted for histological grade. Pretreatment T stage was derived from cT and from imaging-based iT. Ki-67 was entered as a continuous variable and scaled per 10-percentage-point increase. Odds ratios are shown with 95% confidence intervals.

**Figure S4. Relationship between TFAP2B and FOXC1 expression in triple-negative breast cancer cases from the METABRIC cohort.**

Each point represents one tumor. The fitted linear regression line and 95% confidence interval are shown for visualization; the association was assessed using Spearman rank correlation.
